# Development of the US English version of the phenylketonuria – quality of life (PKU-QOL) questionnaire

**DOI:** 10.1186/s12955-017-0620-1

**Published:** 2017-03-09

**Authors:** Elaina Jurecki, Amy Cunningham, Vanessa Birardi, Grégory Gagol, Catherine Acquadro

**Affiliations:** 1 BioMarin Pharmaceutical Inc., 770 Lindaro Street, San Rafael, 94901 CA USA; 2 Hayward Genetics Center SL-31, Tulane University School of Medicine, 1430 Tulane Avenue, New Orleans, 70112 LA USA; 3 Mapi Language Services, 27 rue de la Villette, Lyon, 69003 France; 4 Mapi Research Trust, 27 rue de la Villette, Lyon, 69003 France

**Keywords:** Phenylketonuria, PKU-QOL questionnaire, Health-related quality of life, Translation, Linguistic validation

## Abstract

**Background:**

Phenylketonuria (PKU) is a rare genetic disorder caused by a defect in the metabolism of phenylalanine (PHE) resulting in elevated blood and brain PHE levels, and leading to cognitive, emotional, and psychosocial problems. The phenylketonuria – quality of life (PKU-QOL) questionnaire was the first self-administered disease-specific instrument developed to assess the impact of PKU and its treatment on the health-related quality of life (HRQL) of patients and their caregivers. Available in four versions (child, adolescent, adult and parent), the PKU-QOL was simultaneously developed and validated in seven countries [i.e., France, Germany, Italy, The Netherlands, Spain, Turkey and the United Kingdom (UK)]. The objectives of our study were to develop and linguistically validate the PKU-QOL questionnaire for use in the United States (US).

**Methods:**

The UK versions served as a basis for the development of the US English PKU-QOL questionnaire. The linguistic validation process consisted of 4 steps: 1) adaptation of the UK versions into US English by a translator native of US English and living in the US; 2) a clinician review; 3) cognitive interviews with patients and caregivers to test the appropriateness, understandability and clarity of the US translations; and 4) two proof-readings.

**Results:**

The adaptation from UK to US English revealed the usual syntactic and idiomatic differences between the two languages, such as differences in: 1) Spelling, e.g., “dietician” (UK) vs. “dietitian” (US), or “mum” (UK) vs. “mom” (US); 2) Syntax or punctuation; and 3) Words/expressions use, e.g., “holidays” (UK) vs. “vacation” (US), or “biscuits” (UK) vs. “crackers” (US). The major issue was cultural, and consisted of using a different terminology to describe PKU treatment throughout the questionnaires. The clinician, with the patients and the caregivers, during the interviews suggested to replace “supplement and amino-acid mixture” or “supplements” with “medical formula.” This wording was later changed to “medical food” to be consistent with the terminology used in current US published guidelines.

**Conclusions:**

The translation of the UK English PKU-QOL questionnaire into US English did not raise critical semantic and cultural issues. The PKU-QOL will be valuable for US healthcare providers in individualizing treatment and managing patients with PKU.

## Background

Phenylketonuria (PKU), or phenylalanine hydroxylase deficiency (PAHD), is a rare autosomal recessive disease, induced by the deficiency of the hepatic enzyme, phenylalanine hydroxylase (PAH) that converts the essential amino acid phenylalanine (PHE) into tyrosine (TYR) [1]. This defect results in increased blood concentrations of PHE and toxic accumulation in the brain, leading to cognitive deficiencies, emotional disturbance and psychosocial disabilities [1, 2]. Current treatment for PKU includes a life-long diet highly restrictive in PHE that excludes high protein foods, and is supported nutritionally with medical foods [3] with the goal of maintaining blood PHE in the range of 120–360 μmol/l [3]. Medical foods for PKU provide the amino acids required for normal growth and development, without PHE or with negligible amounts of PHE, and include conditionally essential TYR and varying quantities of carbohydrate, fat, vitamins, and minerals [4, 5]. Pharmacological treatment with sapropterin dihydrochloride (KUVAN® BioMarin Pharmaceutical Inc., Novato, CA) is to date the only Food and Drug Administration (FDA) approved medication indicated for the treatment of PKU in conjunction with a PHE-restricted diet in individuals with tetrahydrobiopterin (BH_4_)-responsive PKU [3, 6]. Although the mechanism of action underlying the BH_4_ therapeutic effect is not entirely understood, it is thought that the primary mechanism-of-action for BH_4_ treatment in PKU is the activation of residual PAH enzyme resulting in increased PHE oxidation to TYR [7, 8]. Research suggests that approximately 50% of PKU patients in the US could exhibit a beneficial response to BH_4_ [9].

The PHE-restricted diet can be burdensome to individuals with PKU and their families, leading to a risk of non-adherence with treatment, especially in adolescents and young adults [10–13]. Obstacles to treatment adherence include time constraints and stress associated with food preparation and record keeping, as well as restrictions imposed on social life. In addition, medical foods and specialty low protein foods may be poorly accepted and can impose a financial burden [11]. Diminishing adherence with age is a global issue. In a study surveying ten European centers, Ahring et al. [14] showed blood PHE control and the percentages of blood PHE concentrations within each center’s local and national target ranges diminished for patients above 16 years of age.

Psychological and neurocognitive problems may be observed in individuals with PKU [15]. In a systematic literature review [16], Enns et al. reported that overall intellectual functioning and specific neuropsychological abilities may be suboptimal in patients treated with diet only and having either high or fluctuating blood PHE concentrations. They described executive dysfunction in working memory, conceptual reasoning, mental flexibility and organizational strategy. Attentional problems leading to negative impacts on academic progress, as well as on self-esteem and emotional development, were noted. Regarding the evaluation of quality of life (QOL), Enns et al. described contrasting results. Out of six studies reviewed, two presented optimal outcomes, i.e., QOL comparable to normal controls [17, 18] and four reported suboptimal results [19–22]. However, more recent studies (published after Enns et al.’s review) suggest that the QOL of patients with PKU is often comparable to that of the general population [23–27]. Common to these studies is the use of generic measures to assess QOL, i.e. questionnaires intended for use irrespective of the underlying disease. This suggests that the observation of normal QOL outcomes might be the result of the lack of specificity of these generic questionnaires, not specifically designed to address the impact of PKU and its treatment on patients’ lives, therefore, failing to assess more subtle problems that may be experienced by individuals with PKU. For instance, in their evaluation of BH_4_ on quality of life, Ziesch et al. [23] used the KINDL (Kinder Lebensqualität), a generic measure of QOL for children (originally developed in German). They noticed that QOL results conflicted with personal reports from children and parents, felt to be related to the use of the KINDL which did not capture aspects that mattered to the patients. As a result, they called for the development of a specific disease-related PKU QOL instrument. Such an instrument should be able to detect decrements in specific domains of the life of patients with PKU as well as potential improvements in these domains due to therapeutic interventions.

The phenylketonuria – quality of life (PKU-QOL) questionnaire was developed to address these issues [28]. This is the first self-administered instrument developed for patients with PKU and their caregivers which assesses PKU symptoms, PKU in general (i.e., physical, emotional, social and overall impacts of PKU), and the impact of treatment. The PKU-QOL exists in four versions, three are age-specific [Child (9–11 years old) PKU-QOL (40 items), Adolescent (12–17 years old) PKU-QOL (58 items), Adult PKU-QOL (65 items)], and one version enables the evaluation of the QOL of children by their caregivers as well as an assessment of the parents’ QOL [Parent PKU-QOL (54 items)]. The four versions share a similar structure, but reflect the specific realities of each population. The PKU-QOL was simultaneously developed and validated in seven countries (i.e., France, Germany, Italy, The Netherlands, Spain, Turkey and the UK).

The objectives of our study were to adapt and linguistically validate the PKU-QOL for use in the United States by healthcare providers to evaluate the QOL of patients with PKU.

## Methods

### Linguistic validation process

The linguistic validation process used to develop the US version of the PKU-QOL was in compliance with the recommendations of the International Society for Pharmacoeconomics and Outcomes Research [29, 30]. The UK questionnaire served as a basis for the development of the US English PKU-QOL.

The process, conducted by a coordinating center (i.e., Mapi Language Services), consisted of 4 steps. The first step was adapting the UK English version of the PKU-QOL questionnaire to US English. The source UK English version of the PKU-QOL was assessed for its suitability in the linguistic and cultural context of the US, and its wording was adapted when needed. The adaptation was performed by a translator native of US English and living in the US. It is important this step be performed in the target country (i.e., US), to make sure the version is adapted to the contemporary context of the country in which it will be used. Discussion with the coordinating center led to the development of a first target US version. A report summarizing the issues encountered and solutions retained was developed. A second step was a clinician review to obtain input from medical experts on specific terminology used. Issues and solutions were discussed with the coordinating center and the in-country translator. The third step consisted of in-depth cognitive individual interviews with patients and caregivers. The objective was to investigate the appropriateness, understandability and clarity of the US PKU-QOL questionnaire. Participants were asked to comment on their understanding of each part of the questionnaire (i.e., instructions, questions and response categories) and suggest alternative formulations where wording was thought to be problematic. Difficulties were scrutinized and solutions were proposed during discussions between the coordinating center and the in-country-consultant. Finally, two proof-readings were conducted by two translators working independently (i.e., the in-country consultant and one translator new to the study).

### Participants

Patients with a formal diagnosis of PKU were recruited by medical experts from sites in the US who agreed to participate in this study. Medical experts were asked to recruit patients of specified ages according to the questionnaire being evaluated. Patients and their caregivers were included if they agreed to participate in the interviews. Patients were not recruited based on their phenotype. Participants in the interviews had to be native English-speaking residents of the US. Participants were not included if they were not able or willing to provide informed consent and were not native US English speakers.

### Analysis

The linguistic validation report was reviewed to identify difficulties and problematic issues, as well as the solutions proposed to overcome them. The types of difficulties were categorized as Cultural (C), Idiomatic (I), Semantic/conceptual (S) or Syntactic (Sy) (Table 1).

**Table 1:** Categorization of translation difficulties

Category	Definition
Cultural (C)	A word or formulation in the original is culturally loaded in the target context due to societal or religious customs (e.g., eating habits in Asian countries). The usage of certain words or phrases based on the culture of a given society may be improper in the target language. *For instance,*, *starchy foods (*e.g., ***potato***, ***bread***, **etc**.*)*, *starchy foods (*e.g.,. ***rice***, ***pasta***, ***chapatti***, **etc**.*)*.
Semantic (S)	Semantics concerns meanings, which are both denotative, i.e. the literal word (lexis), and connotative, namely the set of cultural and/or subjective associations implied by a word in addition to its literal explicit meaning. This category includes lexical differences. *For instance*, *meet your* *** responsibilities***, *meet your* *** duties***, *meet your* *** obligation*** *s*.
Idiomatic/pragmatics (I)	The practicalities of how a language is used in its everyday context are different between the source and target language. For example, one language may have more social registers than another (there are a number of different forms of addressing a person in Japanese, whereas English may only have one) and the idiosyncrasies of one language (repetitions, focus on particular words, use of idiomatic expressions, etc.) may not be found in another. *For instance*, ***I feel*** * downhearted and* *** blue***,*** I feel*** * down and* *** sad***.
Syntactic/grammar (Sy)	Syntactic difficulties correspond to specific aspects related to sentence structure, grammar, punctuation. The structure and grammar of the source and target language may diverge. For example, there is no grammatical form for the past tense in Tagalog. *For instance*, *How flexible* *** have you been finding*** *…?* * How flexible* *** have you found*** *…?*

## Results

### Participants

Interviews were conducted with 15 patients and 5 caregivers of patients with PKU (Table 2). The US versions of the PKU-QOL were administered as follows: Child PKU-QOL to five children; Adolescent PKU-QOL to five adolescents; Adult PKU-QOL to five adults; and Parent PKU-QOL to five parents of the children/adolescents already recruited to test the children and adolescent versions.

**Table 2:** Demographic characteristics of the cognitive interview participants

	Participants*			
Characteristics	Child	Adolescent	Adult	Parent
Age in years: Range (mean)	9–11 (9.8)	12–17 (14.8)	19–36 (28.4)	31–56 (41)
Gender: males/females	2/3	4/1	2/3	2/3
Level of education	4^th^–6^th^ grade (two aged 9 in 4^th^ grade; two aged 10 in 5^th^ grade; one aged 11 in 6^th^ grade)	6^th^–11^th^ grade (one aged 12 in 6^th^ grade; one aged 14 and one aged 15 in 8^th^ grade; one aged 16 in 9^th^ grade, one aged 17 in 11^th^ grade)	11^th^–12^th^ grade (highest obtained)	11^th^–12^th^ grade (highest obtained)

*n = 5 for each population

The general impression reported was favorable. The questionnaire on the whole was reported to be clearly worded and easy to understand. The instructions, as well as the response choices, were found to be straightforward and free from ambiguity. For each item, respondents had no difficulty in choosing their answer.

### Adaptation issues

The adaptation from UK to US English revealed the usual semantic (S), syntactic (Sy) and idiomatic (I) differences between the two languages, such as differences in:

1. Spelling (S): i.e., “dietician” (UK) vs. “dietitian” (US); “mum” (UK) vs. “mom” (US).
2. Syntax or punctuation (Sy): i.e., “I was so angry I wanted to hit something or someone” (UK) vs. “I was so angry that I wanted to hit something or someone” (US); ‘X’ (UK) vs. “X” (US).
3. Words/expressions use (S/I): i.e., “holidays” (UK) vs. “vacation” (US); “please tick the box” (UK) vs. “please check the box” (US); “Following are…” (UK) vs. “Below there are…” (US); “biscuits” (UK) vs. “crackers” (US); “filling in” (UK) vs. “filling out” (US).

At a cultural level, date format i.e., “day/month/year” (UK) was replaced by “month/day/year” (US). The terminology to describe PKU treatment throughout the questionnaires was changed in order to be fully understood by the patients and caregivers. During the interviews, the clinicians, patients and caregivers, suggested simplification of the terminology by replacing “supplement and amino-acid mixture” or “supplements” with a single expression, i.e., “medical formula.” This wording was later changed to “medical food” to be consistent with the terminology used in current US published guidelines [3, 5].

In contrast, words involving feelings (e.g., angry, happy, sad, afraid, bad, shy, embarrassed, left out, irritable, fussy, aggressive, anxious, or moody), symptoms (e.g., headaches, tired) or behavior (e.g., to drink, to eat, to cook) were not changed.

## Discussion

The adaptation of the PKU-QOL questionnaire from UK English to US English did not reveal major semantic or cultural issues. Participating patients with PKU and their caregivers provided input essential to adaptation of the PKU-QOL questionnaire so that each component could be easily understood by the US target population. The PKU-QOL questionnaire was well accepted by the participants of the study, which supports the assumption that concepts assessed and identified during the development of the original PKU-QOL questionnaire [28] were equally relevant to the US patients and their caregivers. We did not recruit patients based on their phenotype. This was a deliberate choice since our intent was not to test the content validity of the US PKU-QOL, but to test how well the patients understood the questionnaire, and whether or not the wording was clear and explicit. However, we acknowledge that, for the future use of the PKU-QOL, disease phenotype is relevant as the degree of dietary PHE restriction is impacted by this. When the PKU-QOL questionnaires are updated in the future, we intend to include an explanatory sentence acknowledging that some patients may not require some treatment components (medical food, special low protein food, etc.), and the questions should be answered with that in mind.

Other than the initial papers on the development and use of the PKU-QOL questionnaire in seven countries [27, 28], published research on cross-cultural perspectives of quality of life of patients with PKU is scarce [31]. Most of the cross-cultural evaluations currently published review diagnostic and management perspectives in various countries [32–35]. The availability of the PKU-QOL questionnaire in eight countries (i.e., France, Germany, Italy, The Netherlands, Spain, Turkey, UK and the US) will encourage cross-cultural research in PKU, and will be the first step to wider development and use in various cultural settings. International studies assessing differences of impact across cultures would be of great interest. They would enable cross-cultural comparisons and improve awareness, tracking, and management of impact on patients with PKU in different cultures, thus providing opportunity for increased support. In addition, the cross-cultural equivalence of the eight language versions of the PKU-QOL questionnaire (due to the use of rigorous cross-cultural methodologies [28] during the development phase), will enable the pooling of data gathered in different countries, and optimize the chance of demonstrating treatment benefit. This will be useful in assessing the impact of standard dietary treatment, pharmacological treatments such as sapropterin, and potential new therapies on clinical outcomes.

## Conclusions

The adaptation of the UK English PKU-QOL questionnaire into US English did not raise critical semantic and cultural issues. The four versions of the PKU-QOL questionnaire are now fully linguistically validated in US English. The PKU-QOL questionnaire will be valuable for US healthcare providers in individualizing treatment and managing patients with PKU. The PKU-QOL questionnaire will allow patients’ perceptions to be assessed and documented as patients age, increasing understanding of the impact of PKU on the QOL of patients and their parents throughout the life cycle. The use of validated tools to assess the impact of standard dietary therapy, pharmacological treatments such as sapropterin, and potential new therapies on clinical outcomes will be valuable in managing patients with PKU in the future, and will encourage collection of data that is consistent across treatment centers.

## Abbreviations

BH_4_: Tetrahydrobiopterin
C: Cultural
FDA: Food and Drug Administration
HRQL: Health-related quality of life
I: Idiomatic
KINDL: Kinder Lebensqualität
PAH: Phenylalanine hydroxylase
PAHD: Phenylalanine hydroxylase deficiency
PHE: Phenylalanine
PKU: Phenylketonuria
PKU-QOL: Phenylketonuria – quality of life
QOL: Quality of life
S: Semantic
Sy: Syntactic
TYR: Tyrosine
UK: United Kingdom
US: United States
